# *Arc1* : a regulator of triglyceride homeostasis in male *Drosophila*

**DOI:** 10.17912/micropub.biology.000945

**Published:** 2023-08-22

**Authors:** Samuel D. Swope, Tyler W. Jones, Kevin N. Mellina, Sarah J. Nichols, Justin R. DiAngelo

**Affiliations:** 1 Pennsylvania State University, Berks Campus, Reading, PA

## Abstract

Achieving metabolic homeostasis is necessary for survival, and many genes are required to control organismal metabolism. A genetic screen in *Drosophila* larvae identified putative fat storage genes including *Arc1* . *Arc1* has been shown to act in neurons to regulate larval lipid storage; however, whether *Arc1* functions to regulate adult metabolism is unknown. *Arc1 ^esm18^* males store more fat than controls while both groups eat similar amounts. *Arc1 ^esm18^* flies express more brummer lipase and less of the glycolytic enzyme triose phosphate isomerase, which may contribute to excess fat observed in these mutants. These results suggest that *Arc1* regulates adult *Drosophila* lipid homeostasis.

**Figure 1. Arc1 mutant Drosophila store excess fat:**
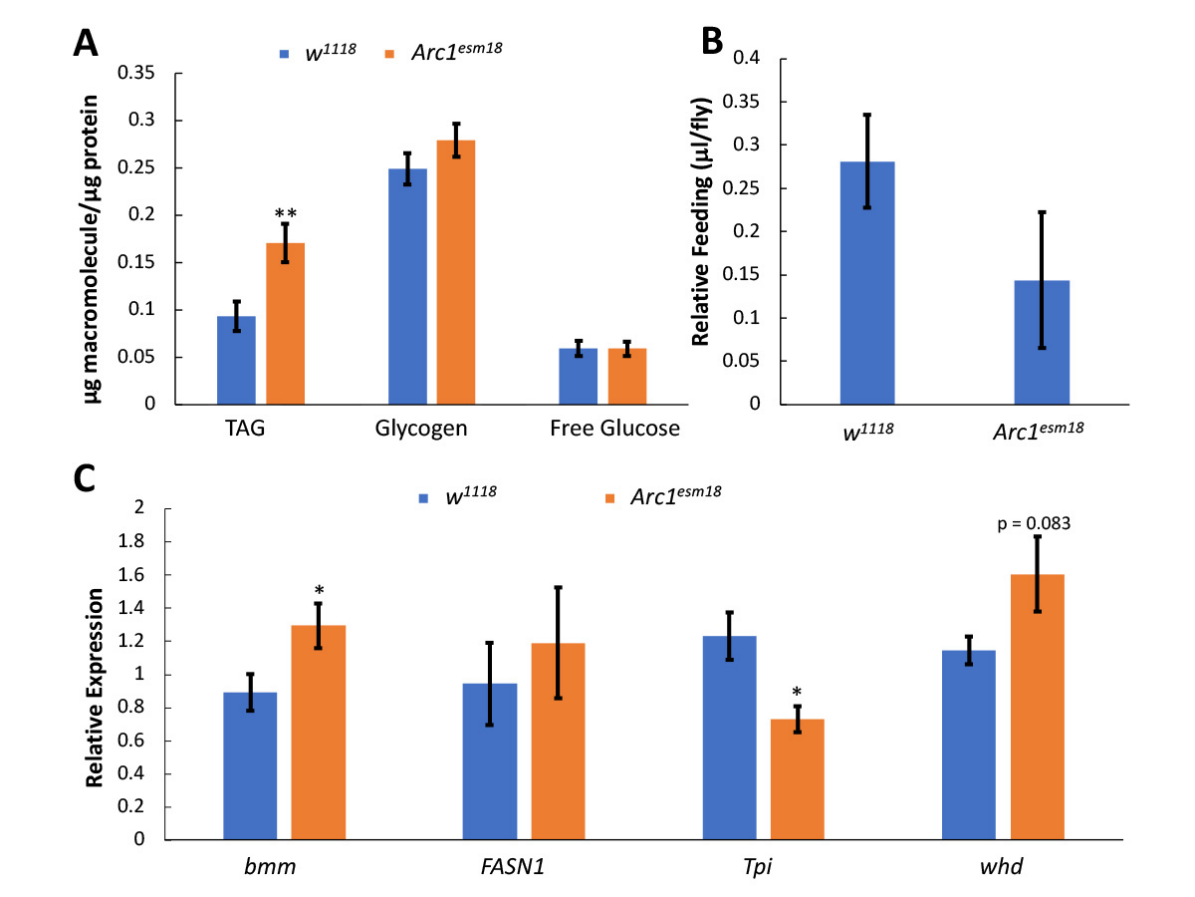
**(A)** Triglyceride (TAG), glycogen, and free glucose concentrations were measured in approximately 1-2 week old male *w ^1118^* and *Arc1 ^esm18^ Drosophila* . Bars indicate average macromolecule concentrations normalized by protein concentrations ± standard error (n=16). **(B)** 1-2 week old male *w ^1118^* and *Arc1 ^esm18^* *Drosophila* were fed 5% sucrose and the amount eaten after 24 hours was measured. Bars represent the average food consumed after 24 hours ± standard error (n=9-12). **(C)** qPCR was performed for *brummer* ( *bmm* ), *fatty acid synthase* ( *FASN1* ), *triose phosphate isomerase* ( *Tpi* ), and *withered* ( *whd* ) from cDNA generated from RNA samples isolated from 1-2 week old male *w ^1118^* and *Arc1 ^esm18^* flies. Expression was normalized by the expression of *rp49.* Bars indicate the average normalized mRNA expression of each gene ± standard error (n=6). * p < 0.05, ** p < 0.01 as determined by unpaired, two-tailed t-test.

## Description

All multicellular organisms require a well-regulated metabolism to survive. When not regulated appropriately, fat stores can accumulate leading to diseases such as obesity and type II diabetes (Singh et al., 2017) . Many genes are required for this to occur; however, not all of them are known. The fruit fly, *Drosophila melanogaster* , provides an excellent system to identify and study genes important for lipid and carbohydrate metabolism. Many metabolic genes present within *Drosophila* are conserved in mammals and *Drosophila* has an adipose-like organ called the fat body that stores and metabolizes lipids similarly to mammalian adipose tissue (Baker and Thummel, 2007; Musselman & Kühnlein, 2018; Heier et al., 2021). To identify genes important for fat storage, a buoyancy-based screen was performed utilizing *Drosophila* larvae (Reis et al., 2010) . This screen identified 66 genes that may be involved in the regulation of fat content and one such gene is the activity-regulated cytoskeleton-associated protein 1 ( *Arc1* ) (Reis et al., 2010) . *Arc1* mutant larvae have increased buoyancy and *Arc1* has been shown to act in specific neurons in the larval brain to regulate fat accumulation (Mosher et al., 2015) . Additionally, *Drosophila* adults with a mutated *Arc1* gene are resistant to starvation (Mattaliano et al., 2007) suggesting a metabolic function; however, whether *Arc1* functions in adult flies to regulate lipid storage is not yet known.

To explore the metabolic functions of *Arc1* in adult flies, triglyceride (TAG), glycogen, and free glucose levels were measured in male control ( *w ^1118^* ) and *Arc1* mutant ( *Arc1 ^esm18^* ) flies. *Arc1* mutant male flies have significantly higher levels of triglycerides than male control flies ( **Fig. 1A** ), consistent with previous work depleting *Arc1* in larvae (Mosher et al., 2015) . It is possible that the increased triglyceride phenotype in *Arc1* mutants is due to increased food consumption. To test this hypothesis, we conducted a feeding assay on male *w ^1118^* and *Arc1 ^esm18^* flies and found that male *Arc1* flies consume the same amount as the controls ( **Fig. 1B** ). This suggests that the excess fat in *Arc1* mutant flies is not due to increasing food consumption.

The triglyceride accumulation seen in male *Arc1* mutant flies may arise from increased lipid synthesis, decreased lipid breakdown, or both. To address this question, we measured the expression of several lipid metabolic enzyme genes in cDNA generated from total RNA isolated from control ( *w ^1118^* ) and mutant *Arc1 (Arc1 ^esm18^* ) flies using reverse transcription-qPCR analysis ( **Fig. 1C** ). We measured the expression of the fatty acid synthesis enzyme gene *FASN1* , the triglyceride lipase gene *bmm* , and the *Drosophila* homolog of the rate limiting reaction in beta oxidation of fatty acids carnitine palmitoyltransferase I (known as *whd* in flies). While there was no change in *FASN1* expression, we observed an increase in *bmm* levels and a trend for an increase in *whd* levels ( **Fig. 1C** ) suggesting that loss of *Arc1* may be increasing the expression of these lipid breakdown genes to compensate for the excess TAG accumulation. Additionally, we measured the expression of the triose phosphate isomerase gene *Tpi,* which encodes an enzyme that interconverts dihydroxyacetone phosphate (DHAP) and glyceraldehyde 3-phosphate (GAP) in glycolysis. We chose to measure *Tpi* levels because Mosher et al. showed increased levels of glycerol-3-phosphate (G3P) and decreased levels of GAP in *Arc1* mutants suggesting a defect in the TPI enzyme. We observed a significant decrease in *Tpi* expression in *Arc1* mutants ( **Fig. 1C** ), suggesting that DHAP is not entering glycolysis as GAP but rather being converted to G3P (a substrate for triglyceride esterification) and may contribute to the triglyceride accumulation phenotype observed in *Arc1* mutants. Together, these results suggest that *Arc1* functions to regulate multiple genes encoding important metabolic enzymes to control lipid homeostasis in adult flies.

Based on our data, the *Arc1* gene appears to have a regulatory role in the storage of lipids in adult *Drosophila* . Our results indicate that male *Arc1* mutant *Drosophila* store more fat than male control flies. This data is consistent with previous studies that found that *Arc1* mutant larvae are more buoyant and have higher levels of fat compared to controls (Reis et al. 2010 and Mosher et al. 2015) . Interestingly, this increase in fat storage is not due to increased feeding as our data also show that these male *Arc1* mutant flies consume the same amount of food as the control flies. Additionally, the increased triglyceride levels shown here is consistent with the starvation resistance phenotype observed previously (Mattaliano et al., 2007) . This previous study noted that *Arc1* mutant flies did not show hyperactive behavior when starved, allowing them to live longer during starvation (Mattaliano et al., 2007) . These starvation resistance and lack of hyperactivity phenotypes could also be due to increased fat storage and further experimentation is necessary to address these possibilities. Moreover, while we only tested triglyceride levels in adult male flies in this study, the increased fat phenotype in the Mosher et al. (2015) study was observed in 3rd instar larvae and the starvation resistance phenotype in Mattaliano et al. (2007) was shown in adult female flies, suggesting that Arc1 may regulate lipid storage similarly at different stages of *Drosophila* development and in both male and female flies; however, additional studies are needed to support this claim.

We also show that the loss of *Arc1* decreases the expression of the glycolysis enzyme triose phosphate isomerase (TPI). This change would increase the levels of DHAP because it cannot be converted into GAP during glycolysis. The extra DHAP could be converted into G3P, a substrate for triglyceride synthesis. This hypothesis is consistent with the decreased levels of GAP and increased levels of G3P observed in *Arc1* mutant larvae (Mosher et al., 2015) . However, whether these changes in GAP and G3P are observed in adult *Arc1* mutants still needs to be shown. In addition, the mechanism of how *Arc1* regulates the expression of genes encoding metabolic enzymes like *bmm* and *Tpi* is not known. Moreover, whether the expression of additional genes encoding metabolic enzymes is altered in *Arc1* mutants is not fully understood. Experiments designed to address these questions are necessary to better our understanding of the metabolic functions of *Arc1* .

In conclusion, we show that the *Arc1* gene plays a role in regulating fat storage in adult *Drosophila* . Portions of *Drosophila Arc1* are conserved in the mammalian *Arc* gene, which is a master regulator of synaptic plasticity in the brain (Shepherd and Bear, 2011; Mattaliano et al., 2007) . Therefore, our results could further our knowledge of the functions of the *Arc* gene in humans, particularly any putative metabolic roles *.* Together, the results of this study expand our understanding of the genes important for regulating lipid homeostasis and could have implications for increasing our understanding of the pathogenesis of metabolic diseases like obesity or type II diabetes in humans.

## Methods

***Fly husbandry.*** Flies were grown at room temperature on sugar-cornmeal-yeast medium (9 g *Drosophila* agar (Genesee Scientific), 100 mL Karo Lite Corn Syrup, 65 g cornmeal, 40 g sucrose, and 25 g whole yeast in 1.25 L water) and 1-2 week old male flies were used in all experiments.

***Triglyceride, Glycogen, and Free glucose Assays.*** Five male *w ^1118^* or *Arc1 ^esm18^ Drosophila* were placed in lysis buffer (140 mM NaCl, 50 mM Tris-HCl, pH 7.5, 0.1% Triton-X with 1X protease inhibitor cocktail (Roche)) and homogenized. A Bradford assay was then conducted using 1X Bradford Reagent (Bio-Rad) as per the manufacturer’s instructions.

The free glucose levels in each sample were measured using Pointe Specific Glucose oxidase reagent (Fisher) as per the manufacturer’s instructions. To measure glycogen levels, each sample was incubated for 2 hours at 37°C in amyloglucosidase (8 mg/mL in 0.2M citrate buffer, pH 5.0 (Sigma)). Pointe Specific Glucose oxidase reagent (Fisher) was added to the digested samples to determine the total glucose concentration in each sample (as per the manufacturer’s instructions.). The free glucose values were then subtracted from the total glucose to determine glycogen concentrations. The triglyceride levels of each sample were determined using Infinity triglyceride reagent (Fisher) as per the manufacturer’s instructions. All free glucose, glycogen and triglyceride values were normalized using corresponding protein concentrations.

***Feeding Assay.*** Food consumption was measured as previously described (Ja et al., 2007) . Briefly, groups of 4 male flies were placed in *Drosophila* vials containing 1% agar. 5 μL capillary tubes were filled with dyed 5% sucrose and the amount of sucrose consumed over 24 hours was measured. A vial with no flies was used as an evaporation control. The distance that the sucrose travelled in each capillary tube was divided by the number of flies in each vial and averaged.

***RNA Isolation and Quantitative PCR.*** Groups of 10 male *w ^1118^* and *Arc1 ^esm18^* mutant flies were homogenized in Trizol (Invitrogen) per manufacturer’s instructions. 5 μg of RNA was then treated with DNase as per manufacturer’s instructions (Ambion). DNase-treated RNA (1 μg) was then reverse transcribed into cDNA using qScript Ultra SuperMix (Quanta Bio) according to manufacturer’s instructions.

Quantitative PCR was performed to amplify the following genes: *bmm, FASN1, Tpi, whd,* and *rp49* (see primer sequences below). Each reaction was prepared using 1 μL of cDNA, 200 nM of each primer, and 1X Perfecta SYBR Green (Quanta Bio) as per manufacturer’s instructions. The qPCR cycling conditions included: 95℃ for 3 min, 40 cycles of 95℃ for 30 sec, 60℃ for 60 sec, and 72℃ for 30 sec followed by a melt curve. The relative expression of each gene was normalized by the corresponding *rp49* expression.

**Table:** 

Gene	Forward (5’ to 3’)	Reverse (5’ to 3’)
*bmm*	ACGTGATCATCTCGGAGTTTG	ATGGTGTTCTCGTCCAGAATG
*FASN1*	CTGGCTGAGCAAGATTGTGTG	TCGCACAACCAGAGCGTAGTA
*Tpi*	ATCAGGCTCAAGAGGTCCAC	GCGTTGATGATGTCCACGAA
*whd*	GCAAGTGCAAATTGAGGAAA	AAGTGCTCCTCACCTTCCAC
*rp49*	GACGCTTCAAGGGACAGTATCTG	AAACGCGGTTCTGCATGAG

***Statistical Analysis.*** Data from each assay was averaged and compared via an unpaired two-tailed t-test with p < 0.05 indicating a significant difference. A p-value of 0.05 or less is indicated by a single asterisk. A p-value of 0.01 or less is indicated by two asterisks.

## Reagents

**Table:** 

**Strain**	**Genotype**	**Available from**
w ^1118^	* w ^1118^*	Bloomington Stock Center #3605
*Arc1* mutant	w[*]; Arc1 ^esm18^	Bloomington Stock Center #37530
